# A two-hit model of alcoholic liver disease that exhibits rapid, severe fibrosis

**DOI:** 10.1371/journal.pone.0249316

**Published:** 2021-03-26

**Authors:** Monideepa Sengupta, Suomia Abuirqeba, Amina Kameric, Aurore Cecile-Valfort, Arindam Chatterjee, Kristine Griffett, Thomas P. Burris, Colin A. Flaveny

**Affiliations:** 1 The Department of Pharmacology and Physiology, Saint Louis University School of Medicine, Saint Louis, MO, United States of America; 2 Center for Clinical Pharmacology at Washington University Saint Louis and The Saint Louis College of Pharmacy, Saint Louis, MO, United States of America; 3 The Alvin J. Siteman Comprehensive Cancer Center at Washington University Saint Louis School of Medicine, Saint Louis, MO, United States of America; University of Navarra School of Medicine and Center for Applied Medical Research (CIMA), SPAIN

## Abstract

Alcoholic liver disease (ALD) is responsible for an average of 50.4% and 44.2%of liver disease deaths among males and females respectively. Driven by alcohol misuse, ALD is often reversible by cessation of consumption. However, abstinence programs can have limited success at curtailing abuse, and the loss of life. ALD, therefore, remains a significant clinical challenge. There is a need for effective treatments that prevent or reverse alcohol-induced liver damage to complement or supplant behavioral interventions. Metabolic syndrome, which is disproportionally prevalent in ALD patients, accelerates the progression of ALD and increases liver disease mortality. Current rodent models of ALD unfortunately do not account for the contribution of the western diet to ALD pathology. To address this, we have developed a rodent model of ALD that integrates the impact of the western diet and alcohol; the WASH-diet model. We show here that the WASH diet, either chronically or in small time-restricted bouts, accelerated ALD pathology with severe steatohepatitis, elevated inflammation and increased fibrosis compared to mice receiving chronic alcohol alone. We also validated our WASH-diet model as an in vivo system for testing the efficacy of experimental ALD treatments. The efficacy of the inverse-agonist SR9238, previously shown to inhibit both non-alcohol and alcohol-induced steatohepatitis progression, was conserved in our WASH-diet model. These findings suggested that the WASH-diet may be useful for in vivo pre-clinical assessment of novel therapies.

## Introduction

Alcohol related liver disease is responsible for about a third of all liver transplants, and 40% of liver-related deaths in the US [1, 2]. In addition to continued efforts to tackle alcohol use disorder (AUD) it is also important to develop pharmacological interventions designed to reduce both alcohol-induced liver disease (ALD) incidence, and mortality. Development of effective ALD therapies, as a result, must rely on rodent models that closely recapitulate ALD pathology. Diet is a major contributing factor to the progression and severity of non-alcohol induced steatohepatitis (NASH). Diet and ALD etiopathology and severity are irrevocably linked [3, 4]. However, pre-clinical rodent models of alcohol-induced steatohepatitis (ASH) do not typically account for the influence of diet. Binge drinking and chronic alcohol misuse are also associated with unhealthy eating habits such as intake of high-calorie processed foods [3, 5, 6]. Obesity and metabolic diseases including diabetes, cardiovascular disease and dyslipidemia are disproportionately prevalent in AUD patients [3]. The likelihood of a prognosis of ALD, steatosis, acute alcoholic hepatitis and cirrhosis is strongly associated with being overweight for at least 10 years [7, 8]. Obesity and alcohol abuse also synergistically enhance the risk of hepatocellular carcinoma [4, 8, 9]. Also, ethanol results in more severe hepatic pathology, dysregulated glucose metabolism and altered lipid metabolism in obese (*ob/ob*) mice [10]. Chronic ethanol exposure also promotes adipose tissue inflammation and altered adipokine expression which influences the innate and adaptive immune systems, and contributes to insulin resistance [4]. All these findings point to the synergistic interaction of alcohol, diet, metabolic disease and ALD. However, it is not completely clear whether the hepatotoxic effects of alcohol consumption are merely additive to NASH, the hepatic symptom of metabolic dysregulation. Also, the combined physiological impact of the dietary factors that promote ASH, in the absence of obesity or insulin resistance, is unclear. The interaction of diet and alcohol-induced liver disease also appears complex when alcohol use is moderate [11]. Surprisingly it has been shown that modest alcohol consumption can suppress NASH which suggests distinct drivers of liver disease in AUD patients and those that occasionally binge drink or utilize alcohol more sparingly [11]. Therefore, it is important to develop refined in vivo models that capture this complex interplay of diet and alcohol use.

The synergistic effects of the western diet, high-calorie binge eating, and chronic alcohol abuse has, as far as we know, not been comprehensively profiled. We decided to develop models of ALD/ASH that chronicles the synergistic effects of chronic alcohol use with the western diet. Here we compared our model to the most well-characterized rodent ASH model, the Lieber-DeCarli (LD) diet, typically used to model acute and chronic alcohol exposure in mice [12, 13]. The LD-diet is nutritionally balanced, therefore, it does not account for the contribution of high fat, refined sugar and cholesterol to ASH. Also, the LD-diet model does not produce hepatic fibrosis, a tenet of ALD in humans. To display hepatic fibrosis, mice on the LD-diet often require a “second-hit” such as a toxicant or inflammatory mediator [14, 15]. Our western ASH diet model (WASH-ethanol diet) was therefore designed to attempt to account for the contribution of diet to ALD and more closely mirror the severe hepatic fibrogenesis observed in humans.

Through our investigations we have found that mice placed on a chronic WASH-diet, showed accelerated progression to hepatomegaly, and steatosis with substantially elevated serum levels of liver transaminases compared to the that of the LD-diet. Alcohol-induced liver pathology including micro- and macro- steatoses, hepatic lipid burden and fibrosis were also severely exacerbated. Surprisingly, we observed that a once-daily high-fat, fructose and cholesterol (HFFC) meal was sufficient to exacerbate ASH progression in ethanol-fed mice that received an otherwise nutritionally balanced diet. This implied that even limited intake of high fat and fructose may be sufficient to exacerbate ALD progression when combined with chronic alcohol use. Lastly, we confirmed that the WASH-diet model can be used to evaluate novel treatments for ALD. We found that the therapeutic activity of the Liver-X-Receptor inverse-agonist SR9238, previously evaluated in LD models [16], was conserved in the WASH-ethanol model. These results collectively reveal that the combination of the western diet and ethanol exposure can be used to evaluate the efficacy of experimental therapies.

## Materials and methods

All experimental protocols were pre-approved by and conducted according to the Saint Louis University School of Medicine Institutional Animal Use and Care Committee (IACUC) guidelines approval number: 2372. Euthanasia was performed by carbon dioxide asphyxiation followed by cervical dislocation.

### Experimental animals

Male 8–10 week old C57BL6J mice (72 total) were purchased from Jackson Laboratories. Mice were single-housed and maintained on 12/12 h light/dark cycle.

### WASH and Leber DeCarli diet

Feeding protocols followed the guidelines established by NIAAA [12]. Mice were randomly assigned to groups and acclimated to single housing conditions for one week then weaned from standard chow onto Leber-DeCarli (BioServ, Frenchtown, NJ) or WASH liquid diets (Table 1). The listed components for each diet were mixed into 1L of water. Mice were gradually weaned onto ethanol starting at 0% and increased by 0.9% every two days for 10 days until a concentration of 4.5% (vol/vol) was reached (Day 0). Body weight was monitored daily. Pair-fed control groups were given equivalent volumes (~20mL) to that of WASH and LD ethanol groups. In pair-fed control diets dextran-maltose was used as a substitute for ethanol-derived calories. Mice were euthanized via CO_2_ asphyxiation followed by cervical dislocation after being on ethanol or control diets for 5 or 7 weeks after Day 0.

**Table 1:** Composition of the high fat, fructose and cholesterol “western” alcohol-induced steatohepatitis diet (WASH) compared to the standard Lieber-DeCarli diet.

COMPONENT	LD-CONTROL (G/L)	LD-ETHANOL (G/L)	WASH-CONTROL (G/L)	WASH-ETHANOL (G/L)
**ETHANOL**	-	4.5% v/v	-	4.5% V/V
**MALTOSE**	-	-	8	**8**
**CHOLESTEROL**	-	-	4.6	**4.6**
**FRUCTOSE**	-	-	34.3	**34.6**
**PRIMEX**	-	-	39.6	**39.6**
**MALTODEXTRIN**	115	24.3	-	**-**
**SUSPENDING AIDS**	6.75	6.75	6.75	**6.75**
**CORN OIL**	8.5	8.5	-	**-**
**OLIVE OIL**	28.4	28.4	-	**-**
**SAFFLOWER OIL**	2.7	2.7	-	**-**
**CASEIN**	41.4	41.4	41.4	**41.4**
**VITAMIN MIX**	2.5	2.5	2.5	**2.5**
**DL-METHIONINE**	0.3	0.3	0.3	**0.3**
**CELLULOSE**	10	10	10	**10**
**CHOLINE BITARTRATE**	0.5	0.5	0.5	**0.5**
**MINERAL MIX**	8.8	8.8	8.8	**8.8**
**L-CYSTINE**	**0.5**	**0.5**	**0.5**	**0.5**

#### SR9238-dosing

Mice (n = 10) were maintained on WASH diets +/- 4.5% ethanol for 2 weeks prior to receiving 30mg/kg SR9238 or 10:10:80 vehicle control (10% DMSO: 10% Tween80: 80% PBS) via intraperitoneal injection once daily for 4 weeks as described [17].

#### HFFC once-daily binge-meal

Mice (n = 10 per group) were placed on the LD diet +/- 4.5% ethanol and received two 400 mg compressed food pellets that comprised all the components of the WASH diet, except water (Table 1); or two 400 mg pellets of standard chow. Each mouse was given two pellets at the beginning of the dark cycle and given 2 h to consume the pellets before being placed in a fresh cage.

### Real-time quantitative PCR

Total RNA was isolated from frozen liver tissue using Tri-reagent and reverse transcribed using the iScript cDNA synthesis kit (Quanta). cDNA was assessed for gene expression using cognate primers via RT-QPCR.

### Body composition analysis

Mice were randomized into weight-matched groups (n = 8) that received the WASH or LD-control diet alone or with ethanol (Table 1). Body composition was analyzed using a Bruker BioSpin LF50 Minispec NMR. Mice were weight before being assessed via NMR. Baseline body composition was analyzed prior to ethanol feeding. Body composition was assessed again at conclusion of the experiment; after mice were on the respective diets 5 or 7 weeks.

### Clinical chemistry

Whole blood was collected following euthanasia via cardiac puncture and serum was isolated by centrifugation in heparin coated tubes at 15000rpm at 4°C. Serum was then assessed for lipid and liver toxicity markers using the Cobas c111 instrument (Roche Diagnostics) and cognate bioassay kits.

### Histology

Liver sections were excised from the left lateral lobes, fixed in 10% neutral buffered formalin overnight and then embedded in paraffin for sectioning. Tissues cut into 10μm-think sections and mounted on slides and stained with hematoxylin and eosin or subjected to histological assays listed below.

#### Masson tri-chrome staining

Paraffin sections of the left lateral lobe were de-paraffinized and hydrated with deionized water and stained with Bouin’s Solution, Wiegerts Iron Hematoxylin and Biebrich Scarlet-Acid Fuchsin as per manufacturer’s instructions (Sigma-Aldrich).

#### PicoSirius Red staining

10μM sections were stained with Pico Sirius Red using the Direct Red-80 kit (Sigma) and assessed for collagen using light microscopy. Collagen staining (Red) was quantified using ImageJ.

#### Immunohistochemistry

FFPE slides were deparaffinized in xylene and rehydrated in ethanol. After performing antigen retrieval using DIVA de-cloaker (Biocare Medical) and blocking with 5% donkey serum, sections were washed and incubated with primary antibodies (anti-TGF-B 1:1000; anti-IL6 1:200) overnight at 4°C. Sections were washed and treated with secondary HRP-conjugate (donkey anti-rabbit 1:300) for 1 hr. Sections were then incubated with DAB substrate until desired staining was achieved (45s-1min) and counterstained with hematoxylin. Slides were dehydrated and mounted prior to imaging

### Immunoblots

Total protein was isolated from flash-frozen liver tissue using RIPA buffer (IL6, TGFB) and run on a 2–10% gradient gel and transferred to a PVDF membrane. For collagen quantification protein lysates were isolated from frozen liver tissue using 0.075M citrate buffer (pH 6) and resolved using 2–8% gradient gel and transferred to PVDF under non-denaturing conditions. Membranes were blocked with 4% BSA and probed with antibodies against TGFB (Abcam, ab92486) IL6 (Santa Cruz Biotechnology: (m-19), sc-1265) HSP90 (Cell signaling, #4874) or COL1A1 (Abcam, ab34710). Protein expression was quantified and normalized to HSP90 using ImageJ. Latent TGFB-L (44kDa) and mature TGFB-M (13kDA) were detected using the same antibody (ab92486). For fold-expression of proteins expression from ethanol fed from mice was normalized to pair-fed controls.

#### BODPIY staining

The right lateral lobes if isolated livers were placed in 4% PFA overnight and then transferred to 10% sucrose in PBS for 2 days followed by 20% sucrose in PBS for another 2 days and finally 30% sucrose in PBS for cryoprotection. These tissues were then embedded in OCT and frozen in 2-methylbutane dry ice bath. 10um sections were floated in 6-well dishes containing 1X PBS and Bodipy 493/503 (Molecular Probes) staining as described previously. Floated sections were stained and mounted on slides prior then counterstained with DAPI.

### Statistical analysis

Significant differences between mean body mass, liver weight to body rate ratios and body composition parameters were all determined using 2-Way ANOVA with multiple comparisons. For body composition analysis, group sample sizes (n = 8) were determined using power set at 0.8 with alpha set to 0.05 to detect differences in mean of 4% and a standard deviation of approximately +/-2.5% resulting in the calculated sample size of n = 8. For mice treated with SR9238 the estimated intergroup difference in mean gene expression was 0.4 with standard deviation of +/-0.3 so the sample size was set to n = 10 for these studies. Clinical chemistry markers, mRNA (RT-QPCR) and protein (western blot, IHC) expression was compared using 1-Way ANOVA or student’s t-test where relevant. *p<0.05, **p<0.01***p<0.001. All graphs were generated and analyzed for significant differences using Prism.

## Results

### Hepatic toxicity is synergistically increased by the combination of dietary fat and fructose with ethanol

**Fig 1:**
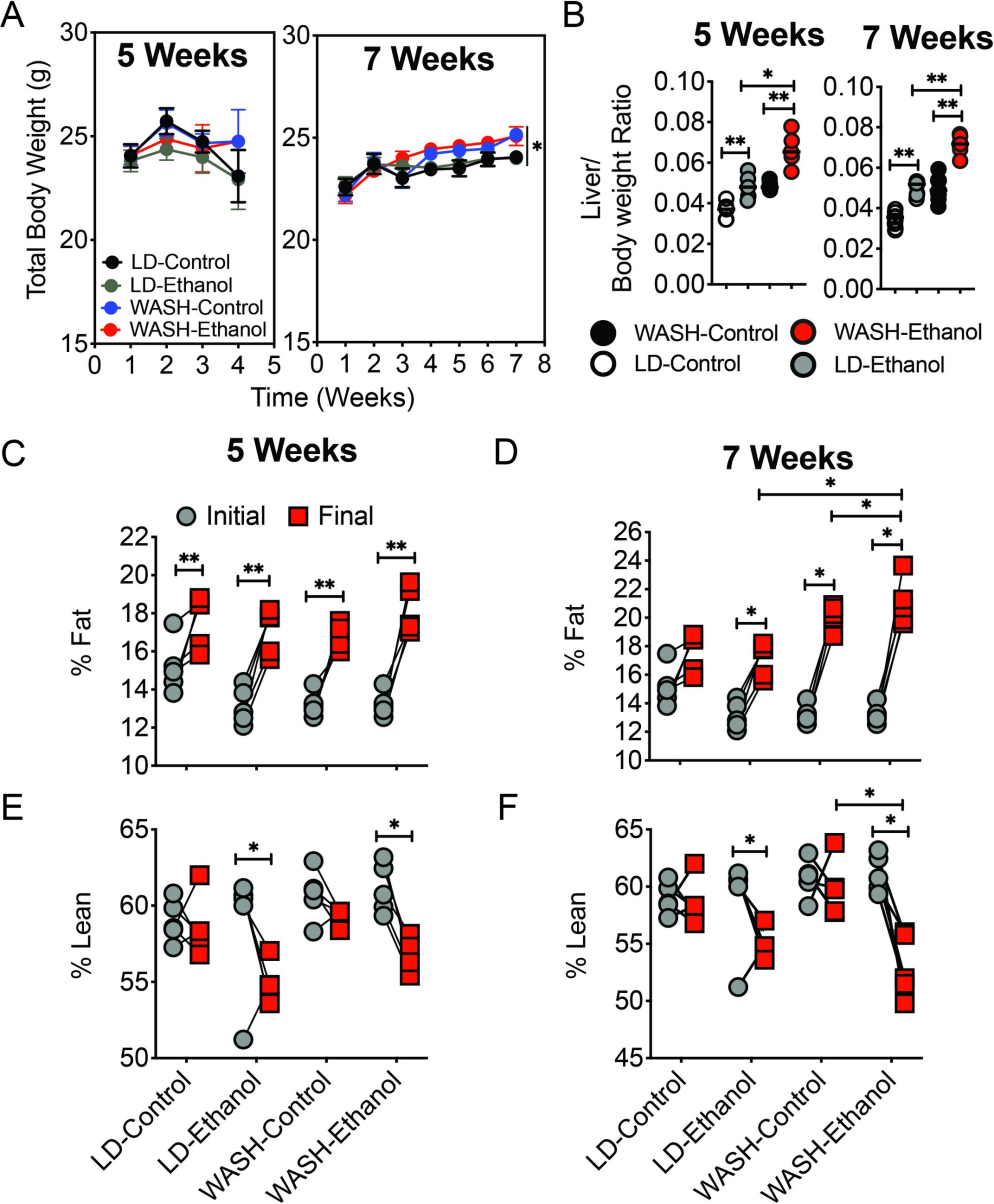
Ethanol combined with fat, cholesterol and fructose promotes hepatomegaly and whole-body adiposity. **A.** Total body weight of mice placed on the Lieber De-Carli (LD) or Western/Alcohol Steatohepatitis diet (WASH) with (LD-Ethanol, WASH-Ethanol) or without 4.5% ethanol for 5 weeks (left panel) or 7 weeks (right panel) **B.** Liver to body weight rations of mice fed the LD and WASH ethanol or cognate control diets for 5 or 7 weeks. **C.** Percent fat mass of mice fed LD and WASH diets for 5 weeks or **D.** 7 weeks **E.** Percentage fat-free lean mice in mice fed the LD and WASH diets for 5 or **F.**7 weeks. Body composition measured using BrükerSpin mini spec rodent NMR. Sample size is n = 8 per group. Means compared using One-Way ANOVA. *p<0.05. +/- standard error of the mean (S.E.M).

In order to capture the effects of diet on ASH pathology we characterized the hepatic effects of a high fat, cholesterol and fructose diet on the backdrop of chronic alcohol intake. Previous groups have studied the effect of obesity of ASH pathology [4, 10, 18–20]. We wanted to probe the contribution of macro-nutrient intake, typical of western diets, on ASH progression prior to the onset of obesity. We formulated the western-ASH (WASH) diet to comprise elevated amounts of cholesterol (4.6 g/L), trans-fat (39.6 g/L) and fructose (34.3 g/L) (Table 1) as well as the calorically equivalent control diet supplemented with maltose, corn, safflower and olive oil as cognate macronutrient controls for fructose and vegetable-derived trans-fat (Primex). We compared the hallmarks of ALD hepatic pathology in mice fed either the standard chow, Lieber-DeCarli (LD) diet or WASH-diet with or without chronic 4.5%(v/v) ethanol for 5 or 7 weeks (n = 8). The average total body weight of all mice in the study were within normal range (22-28g) for 8–10 week-old mice (Fig 1A). There were no significant differences in body weight in mice placed on the LD or WASH-Diet after 5 weeks (Fig 1A). However, mice receiving the WASH-diet showed an increase in total body weight after 7 weeks compared to LD-diet mice (Fig 1A right panel). The liver to body weight ratios, a marker of hepatomegaly, in mice receiving the WASH-diet plus 4.5% ethanol was significantly greater than that of LD-ethanol mice (Fig 1B). This indicated that liver injury in WASH-ethanol diet mice was more pronounced. NMR assessment of body composition showed that all mice displayed a shift toward adiposity regardless of diet (Fig 1C) after 5 weeks. However, at 7 weeks while the shift in adiposity was conserved, mice fed WASH-ethanol displayed a significant increase in percentage fat mass compared to that of LD-ethanol animals (Fig 1D). Similarly, ethanol-fed groups showed a reduction in lean mass regardless of diet at both 5 and 7 weeks (Fig 1E and 1F). Notably ethanol did not significantly reduce lean mass in WASH-ethanol mice compared to LD-ethanol mice (Fig 1E and 1F). The observations suggested collectively that the lipid and fructose burden of the WASH-ethanol diet had a additively deleterious effect on liver injury and whole body adiposity that exceeded that of the LD diet.

### Serum cholesterol, triglycerides and markers of liver injury are significantly elevated by the WASH-ethanol diet

**Fig 2:**
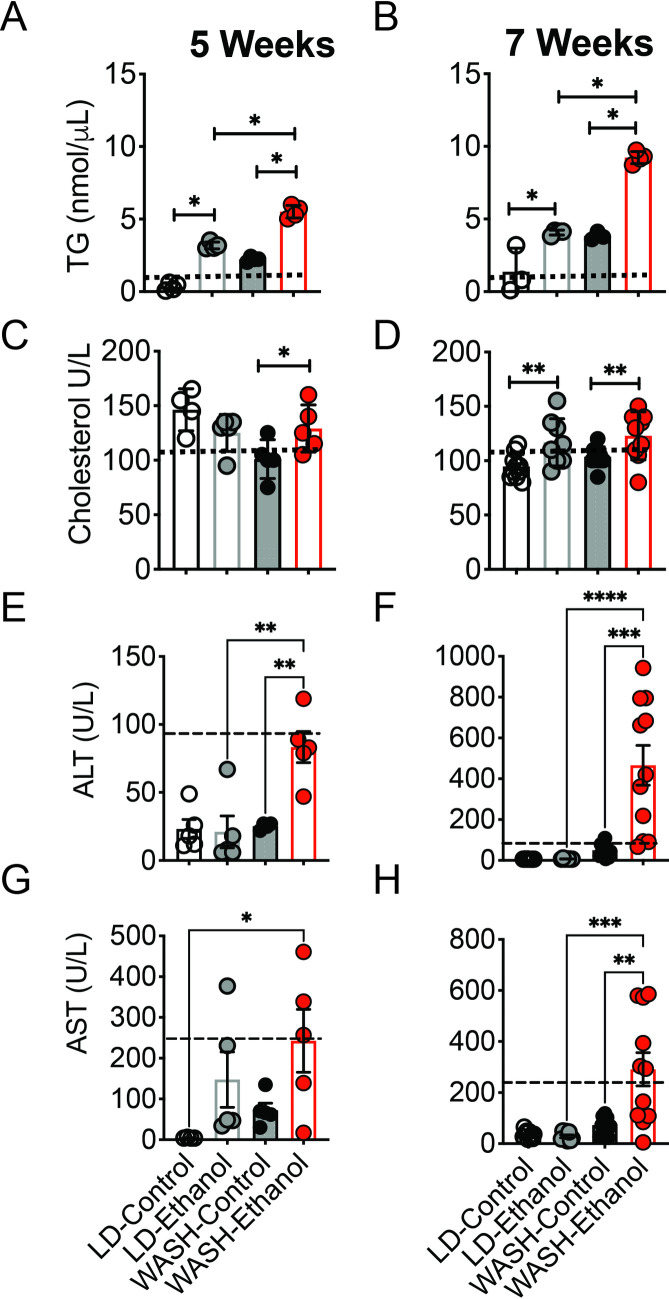
Ethanol induced liver toxicity is exacerbated by the WASH diet. **A.** Serum triglyceride (TG) levels in mice placed on the LD and WASH diets +/- ethanol for 5 or **B.** 7 weeks. **C.** Total circulating cholesterol in mice fed the WASH or LD diets+/- ethanol for 5 or **D.** 7 weeks **E.** Serum alanine amino transferase (ALT) levels in mice fed the WASH and LD diets +/- ethanol for 5 weeks or **F.** 7 weeks. **G.** Serum aspartate amino transferase (ALT) levels in mice fed the WASH and LD diets +/- ethanol for 5 weeks or **H.** 7 weeks. Serum was isolated from cardiac blood. Serum levels of lipids and proteins were determined using a Cobas C11 clinical chemistry bioanalyzer (Roche Diagnostics). (n = 8 mice per group). Mean levels were compared using One-Way ANOVA. * p<0.05, **p<0.01 ***p<0.001 +/- S.E.M.

We evaluated the changes in lipid metabolism and determine whether clinical markers of liver injury correlated with our previous findings. Liver triglycerides (TGs) were significantly higher in WASH-ethanol mice at 5 and 7 weeks compared to LD-ethanol mice (n = 8) (Fig 2A and 2B). At 5 weeks, plasma cholesterol was only increased in WASH-ethanol mice compared to WASH-controls, and relative to LD-diet mice (Fig 2C). Similarly, at 7 weeks both LD- and WASH-ethanol groups showed significantly increased cholesterol compared to their cognate controls (Fig 2D). At 5 weeks circulating levels of alanine amino-transferase (ALT) in WASH-ethanol mice exceeded that of WASH-controls but LD-ethanol mice showed no significant increase (Fig 2E). Two weeks after LD-ethanol mice did not display elevated ALT. However, WASH-ethanol mice showed a robust rise in ALT at 7 weeks (Fig 2F). Conversely, aspartate aminotransaminase (AST) were significantly elevated in mice receiving WASH-ethanol compared to pair-fed controls and LD-ethanol mice at 7 weeks but not after 5 weeks (Fig 2G and 2H). These results suggest that the WASH-ethanol diet produces accelerated liver toxicity and pronounced disruption of hepatic lipid metabolism that may contribute to more severe liver pathology compared to the LD-ethanol diet.

### The WASH-ethanol diet produces pronounced hepatic steatosis and accelerated lipid accumulation

**Fig 3:**
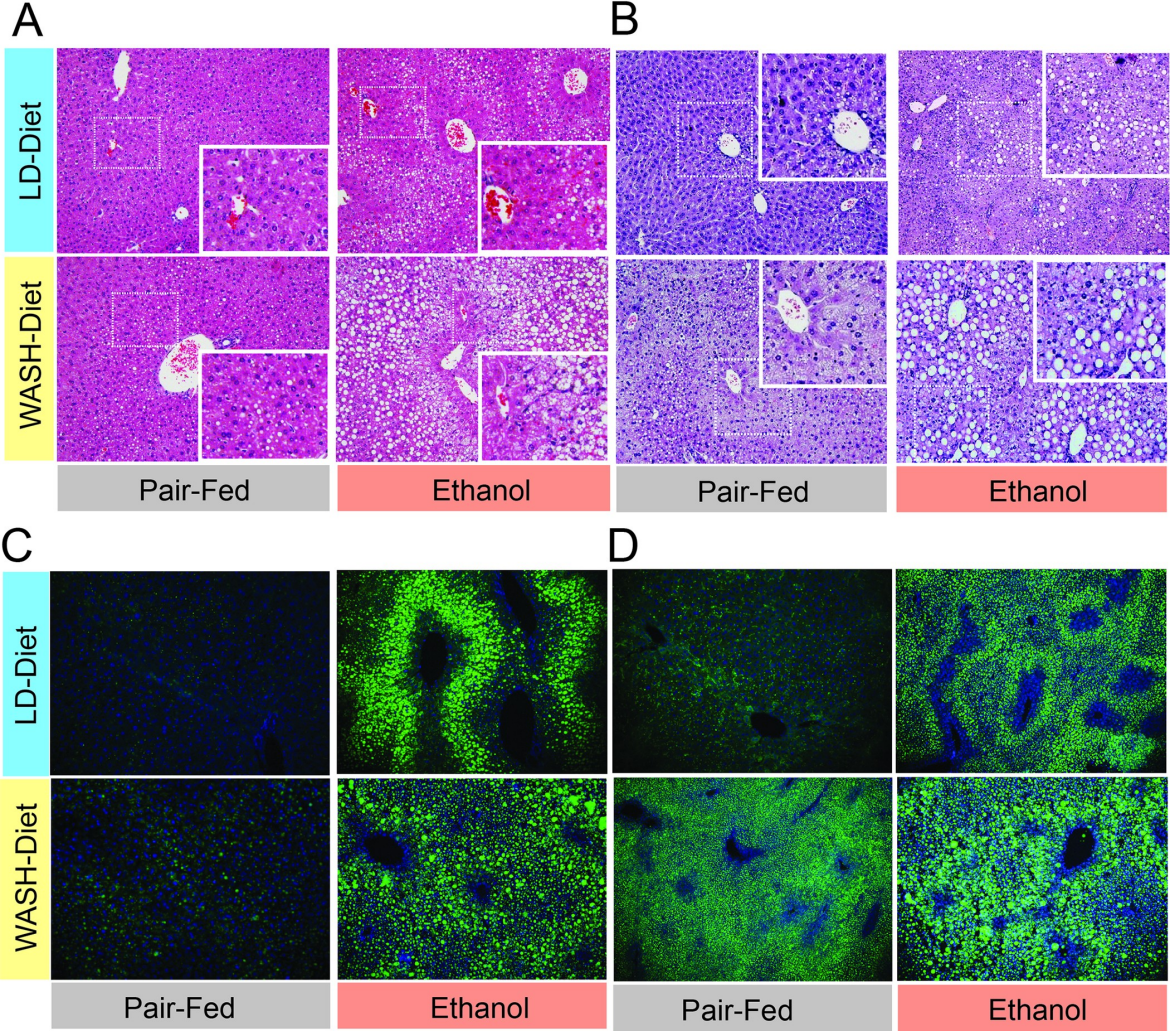
Hepatic lipid content is elevated in mice fed the WASH-ethanol diet. **A.** Hematoxylin and eosin (H&E) staining of liver sections from fed the LD and WASH diets +/- ethanol for 5 weeks **B.** 7 weeks **C.** BODIPY neutral lipid staining of liver samples from mice receiving the LD and WASH ethanol diet +/- ethanol for 5 or **D.** 7 weeks. Representative images from sections analyzed from 8 mice (n = 8). Left (H&E) or right (BODIPY) lateral lobes were stained as described in the materials and methods section.

To determine whether the WASH-ethanol and LD-ethanol differentially influenced liver pathology, we performed hematoxylin eosin (H&E) staining on liver sections from mice exposed to these diets +/- ethanol. At 5 weeks the WASH-control mice displayed minor micro-steatosis that partly extended into the intermediary region between to lobule arteries and veins (Zone 2) (Fig 3A). As expected in the LD-ethanol group there was visible micro and macro-steatoses that extended into the intermediate region (Zone 2) (Fig 3A). LD-controls displayed restricted micro-steatosis that did not extend past the hepatic lobule periphery (Zone 1) into the intermediate zone. In contrast, WASH-ethanol mice displayed significantly more severe liver morphology with macro and micro-steatosis extended into the periphery of the portal vein (Zone 3) (Fig 3A). At 7 weeks WASH-ethanol diet caused more pronounced and extensive macro-steatosis (Fig 3B). Liver sections were also assessed for lipid content using BODIPY neutral lipid staining. The combination of the WASH diet and ethanol synergistically increased hepatic dyslipidemia compared to pair-fed controls and LD-ethanol mice at 5 weeks that became more sever at 7 weeks (Fig 3B and 3C). These results highlight that the high fat/fructose and ethanol had synergistic effects on liver pathology and lipid metabolism when combined with ethanol.

### Combined dietary fat, fructose and ethanol produces extensive liver fibrosis

**Fig 4:**
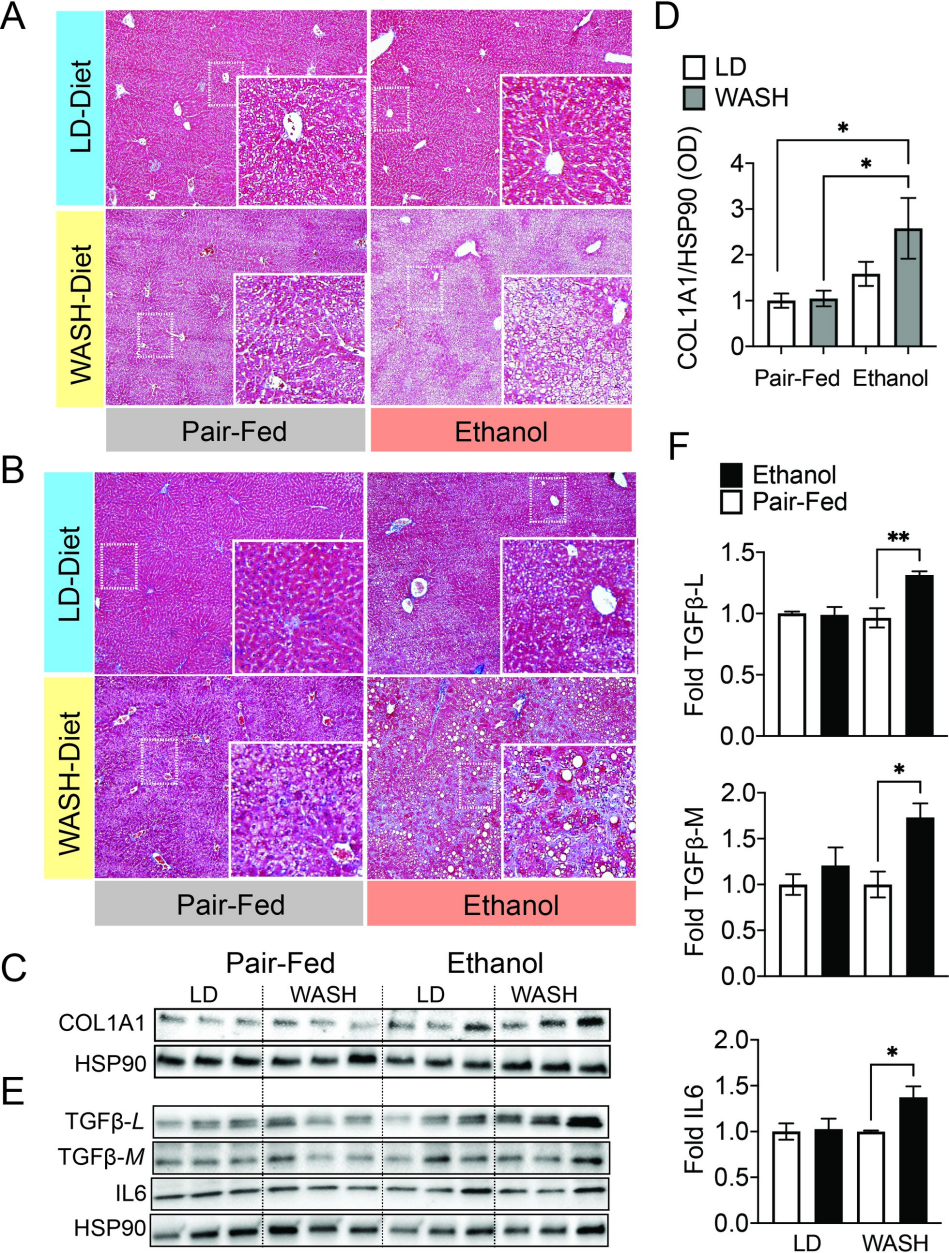
WASH-ethanol diet fed mice show worsened liver pathology and amplified fibrosis. **A.** Masson Trichome staining of liver sections isolated from mice exposed to the WASH or LD diet +/- ethanol for 5 or **B.** 7 weeks. Trichrome staining was performed on left lateral lobe liver sections that were formalin fixed paraffin embedded. Representative image (n = 8). **C.** Immunoblot showing expression of latent and mature (TGFB-L and TGFB-M respectively), IL6 and HSP90 in mice placed on their respective diets for 7 weeks. **D.** Fold protein expression of TGFB-L, TGFB-M and IL6 in mice placed on the LD or WASH control or ethanol diets for 7 weeks. Target proteins normalized to HSP90 and represented as fold increase relative to LD- and WASH-Diet Pair-fed controls. OD quantified in ImageJ. **E.** Immunoblot showing collagen expression (COL1A1) in LD and WASH control versus ethanol-fed mice at 7 weeks. **F.** Quantified expression of COL1A1 normalized to HSP90 in the samples shown in E. Means compared using student’s t-test *p<0.05 **p<0.01. +/- S.E.M.

One limitation of the LD-ethanol model is that it does not intrinsically lead to hepatic fibrosis which is a hallmark of human ASH. We therefore assessed collagen deposition using Masson Trichrome Staining in the livers of mice fed the WASH and LD diets +/- ethanol. At 5 weeks neither mice placed on the LD-ethanol or WASH-ethanol diet displayed fibrosis. Interestingly, mice receiving the WASH-ethanol diet had extensive fibrosis at 7 weeks, based on Masson tri-chrome staining, which was not observed in other groups (Fig 4A and 4B). Hepatic collagen protein (COL1A1) levels was also elevated in WASH-ethanol compared to WASH-control mice (Fig 4D). In addition, hepatic protein levels of the inflammatory factors IL6 and TGFB were only increased in mice placed on the WASH-ethanol diet for 7 weeks (Fig 4E and 4F). These findings imply that the fat and fructose in the WASH diet may be sufficient “second-hits” for effectively driving hepatic inflammation and fibrinogenesis.

### Restricted feeding of high fat, cholesterol and fructose accelerates chronic alcohol-induced liver damage

**Fig 5:**
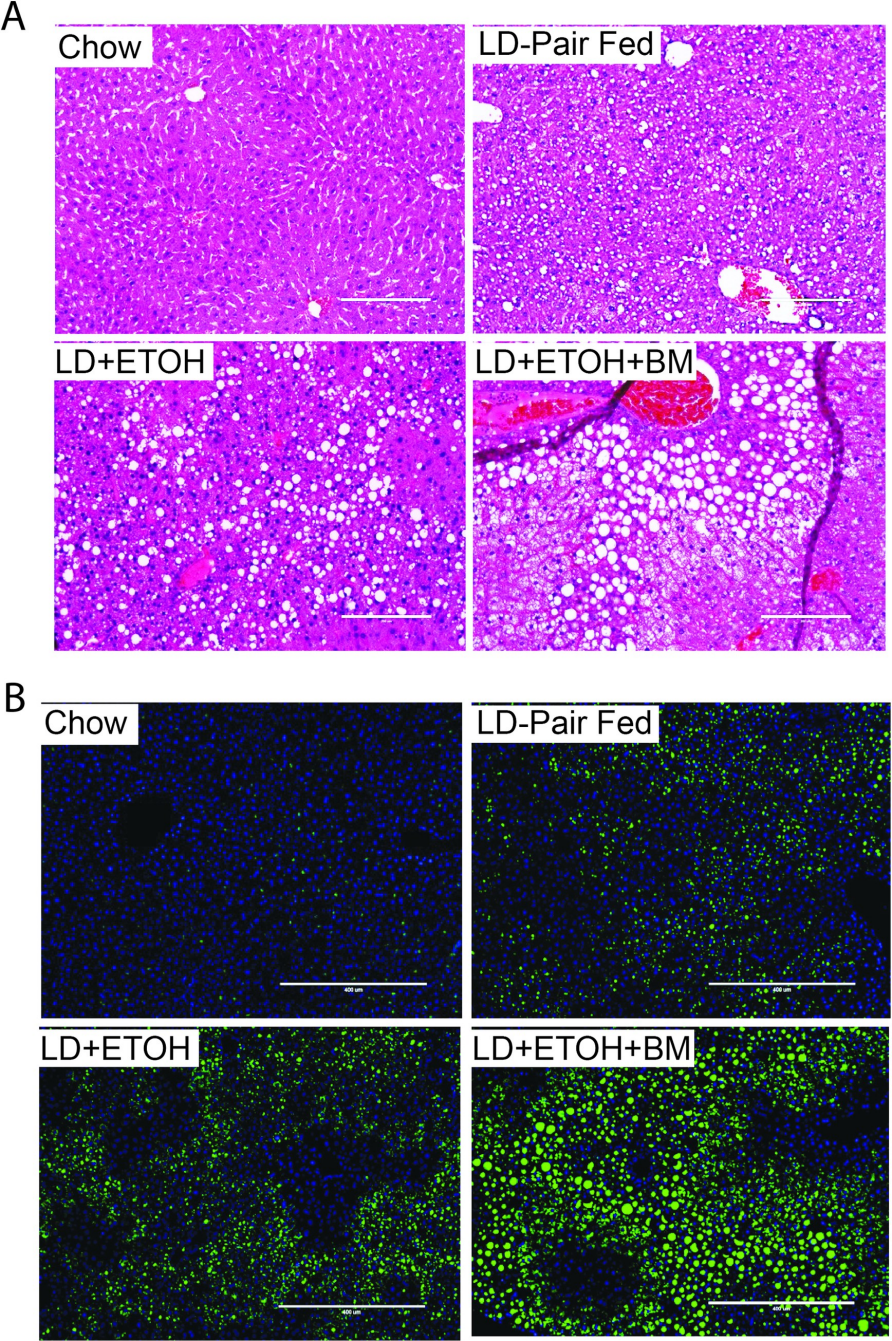
Once-daily high fat, fructose and cholesterol meals (HFFC) significantly altered ethanol induced liver pathology. **A.** H&E staining of liver sections of mice fed the LD-diet +/- ethanol with or without a once daily meal of two 400mg high fat fructose and cholesterol (HFFC) or control pellets between 1200 and 1400 h daily. **B.** BODIPY-neutral lipid staining of liver sections showing the lipid content of the livers from mice on the LD-diet +/- ethanol with HFFC pellets or control chow pellets. Representative slides (n = 10). Mice we allowed to ingest HFFC pellets of chow pellet controls for 2 h before being transferred to a fresh cage.

Considering the metabolic burden of chronic high fat/fructose intake in the WASH diet we decided to explore whether restricted bouts of once daily high fat/fructose meals was sufficient to exacerbate the effect of chronic ethanol intake on liver pathology. We formulated a, high-fructose trans-fat and cholesterol meal (HFFC), which comprised the same macronutrients as the WASH diet (Table 1). We then designed a study to explore the effect of restricted once daily intake of calorie-rich, but micronutrient-deficient, meals on ALD progression. 8-week-old, C57BL6/J male mice were placed on the LD-ethanol or LD-control diet and given two 400mg HFFC or standard chow pellets once a day during a 2 h time window for 4 weeks. Interestingly, mice receiving the HFFC pellets displayed more severe liver pathology with accelerated development of steatosis (Fig 5A). These HFFC meals also stimulated greater hepatic lipid accumulation compared to pair-fed controls (Fig 5B). There results suggested that even limited daily dietary intake of high fat, cholesterol and fructose meals can contribute to ALD progression when combined with high alcohol intake.

### The WASH model can be used to evaluate experimental therapies for ALD

**Fig 6:**
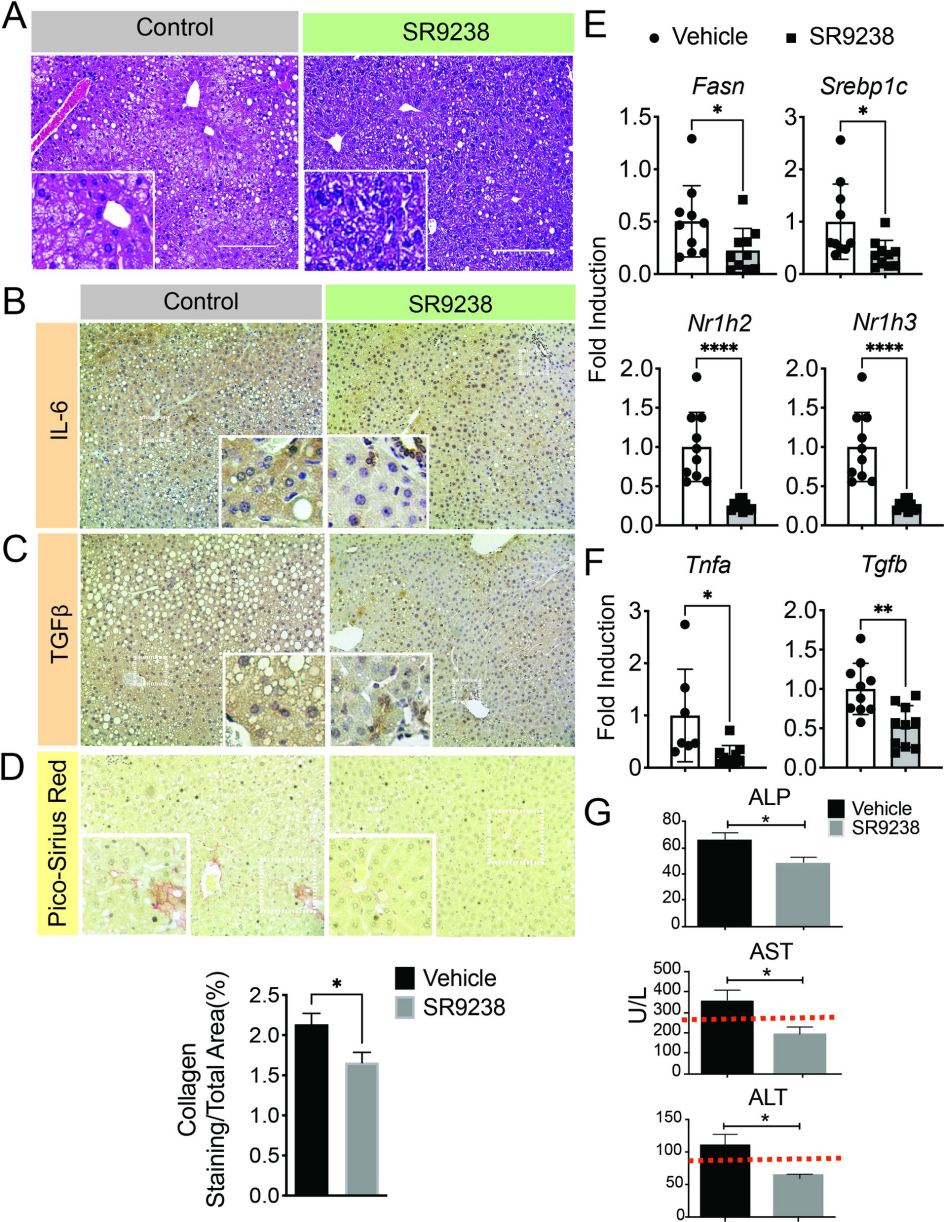
The WASH-ethanol diet model can be used to evaluate the effect of novel drugs on liver, toxicity, pathology and fibrogenesis. **A.** H&E staining of liver sections of mice (n = 10) placed on the WASH-ethanol diet and dosed with 30mg/kg SR9238 or vehicle control (10:10:80 DMSO:Tween80:PBS) Representative images (n = 10). Mice were placed on the WASH diet +/- ethanol for 2 weeks before dosing began. SR9238 or vehicle was administered via i.p. once daily for 4 weeks. **B.** Immunohistochemistry (IHC) showing expression IL6 in the livers of vehicle versus SR9238 dosed mice. Representative images (n = 10). **C.** IHC expression of TGFB in vehicle and SR9238 treated mice. Representative images (n = 10). **D.** PicoSirius Red staining showing hepatic collagen deposits in livers of mice treated with SR9238 or vehicle. (Lower panel) Percent area of collagen positive staining in WASH-diet + ethanol-fed mice treated with SR9238 or vehicle. PicoSirius Red staining was quantified using image J. Representative image (n = 10) **D.** Liver-transaminases (ALP, AST and ALT) in mice fed the WASH-ethanol diet and dosed with SR9238. Liver transaminase levels were quantified using the Cobas C11 bioanalyzer and cognate assay kits (n = 8). **E.** RT-QPCR showing expression of the LXR isotypes *Nr1h3*, *Nr1h2*, *Fatty-acid synthase* (*Fasn*) and *Sterol-regulatory element binding protein-1c (Srebp1c)*. **F.** Hepatic mRNA expression of *Tnfa* and *Tgfb* in vehicle and SR9238 dosed mice. Total mRNA was isolated from flash frozen liver sections and gene expression was quantified using cognate primers. Mean values were compared using student’s-t test. *p<0.05 +/- S.E.M.

We have previously shown that the inverse agonist of the Liver-X-Receptor SR9238 potently reduces hepatic lipogenesis and slows the progression of NASH and ALD [16, 17]. To determine whether the WASH model was a useful model system for evaluating the efficacy of experimental drugs we tested the efficacy of SR9238 in mice receiving the WASH-ethanol diet or pair-fed controls (Fig 6 and S1 Fig). Mice were placed on the WASH diet for 2 weeks then dosed with 30mg/kg SR9238 via intraperitoneal (i.p.) injection once a day for 4 weeks. As expected, based on previous investigations [16] liver pathology was significantly improved in mice receiving SR9238 (Fig 6A). In WASH-ethanol mice SR9238 reduced hepatic IL6 and TGFB protein expression (Fig 6B and 6C). Pico Sirius Red staining also revealed that SR9238 reduced hepatic fibrosis in WASH-ethanol mice (Fig 6D). Although the hepatic fibrosis observed was not as severe as that of mice placed on the diet for 7 weeks (Fig 4B). Improved liver pathology coincided with a significant reduction Liver-X-Receptor (LXR) target-gene expression including enzyme and transcriptional mediators of hepatic lipogenesis such as fatty acid synthase (*Fasn*) and sterol-regulatory element binding protein 1c (*Srebp1c*) and LXR (*Nr1h2* and *Nr1h3*) (Fig 6E). Expression of *Tnfa* and *Tgfb* was also similarly downregulated in SR9238 treated mice indicating that LXR inhibition was able to mitigate hepatic inflammatory signaling at a transcriptional level (Fig 6F). In tandem with these findings liver toxicity markers were reduced to normal levels in mice treated with SR9238 (Fig 6G). These findings confirmed that SR9238 displayed comparable therapeutic efficacy in the WASH as observed in the classic LD models and LD-ethanol binge models [16]. These results reveal that the WASH model may be a useful model for exploring the therapeutic activity of newly developed pharmacological agents.

## Conclusions

In this study we developed an ALD model that combines chronic alcohol intake with the metabolic burden of the western diet to quantify the combined effect high fat, cholesterol, fructose and alcohol abuse. We show here that our WASH diet significantly exacerbates ALD pathogenesis and produces pronounced steatohepatitis, inflammation and fibrosis compared to that the LD-ethanol diet. Further we highlight that even if only limited to once daily intake, feeding mice on the LD-base diet high fat and fructose meal pellets promoted more severe hepatic dyslipidemia compared to the LD-ethanol diet alone. This observation was particularly interesting considering consumption of fast food rich in saturated fat and fructose is commonly associated with binge drinking. There are no reported studies that probe the combined biological effect of binge drinking and fast food intake. Whether the HFFC model could be used to map the acute or long term physiological consequences of ingesting fast food after binge drinking is unclear and should be explored.

The development of the ALD models in mice such as the Lieber-DeCarli diet have allowed investigators to explore the mechanisms that drive ALD. Classic LD models, however, are unable to accurately recapitulate ALD in humans. Specifically, LD models do not develop fibrosis and never progress to cirrhosis. Liver fibrosis in LD-mouse models is often limited and requires a “second-hit” such as carbon tetrachloride or inflammatory insult (e.g., IL6) in order to produce detectable hepatic fibrosis. Other approaches utilize forced-feeding or alcohol binges which require surgical procedures that come with increased risk of acute alcohol toxicity or surgical complications that contribute to increased mortality in experimental animals [12]. Conversely while the role of dietary fat intake is known to contribute to ALD pathogenesis and mortality in humans, few rodent models employ dietary fat as a secondary insult. However, while we observed increased fibrinogenesis and inflammation, it is important to note that the most pronounced synergistic effect of alcohol and high fat was on steatosis in the WASH model described here. This suggest that disrupted hepatic lipid metabolism may be an important pathophysiological component to ALD that should be leveraged in rodent ALD diets.

Our collective understanding of the toxic effects of alcohol on hepatic function and physiology is growing. However, there are few treatments other than behavioral changes such as abstinence available to chronic alcohol abusers, as many patients are ineligible for liver transplant. In some cases, advanced liver disease can continue to progress despite abstinence. Considering the formidable psychological challenge and the high rate of recidivism for alcohol addiction, hepatoprotective treatments are going to become increasingly important for addressing these clinical challenges. Lastly hepatoprotective drugs may provide reprieve and valuable time for patients struggling with alcohol addiction. Studies such as this that attempt to improve on the current rodent models of ASH will lead to more refined targeted treatments that could help address these clinical challenges long term.

As mentioned, it is difficult to produce fibrosis in mouse models of ALD without the utilization inflammatory mediators or toxic factors. Cirrhosis and hepatocellular carcinoma, the penultimate causes of mortality in humans has not been documented in any mouse ethanol diet models. In humans ALD is typified by hepatic fibrosis that eventually progresses to cirrhosis which is a precursor to malignant disease. In this study pronounced fibrosis was induced in the WASH-ethanol model in 7 weeks. Whether mice fed the WASH-ethanol diet can be induced to develop cirrhosis or carcinoma is unknown and should be the subject of future study. It also remains to be determined whether enhanced dietary lipid burden in other mouse models of hepatic fibrinogenesis, such as choline-deficient diets, can be modified to produce not only severe fibrosis, but potentially cirrhosis and carcinogenesis. Importantly in these potential models the ethical concerns of exposing mice to ethanol for protracted periods of time must be weighed against the import and impact of a mouse model of alcohol-induced cirrhosis and cancer.

Dyslipidemia and adipogenesis are largely associated with the development and severity of ALD. Interestingly, brown adipose tissue activation has been shown to be hepatoprotective in models of ALD in contrast to white adipose tissue [21]. The mice in our studies did not become obese but displayed high adiposity (WAT) (Fig 1). However, we did not quantify the relative composition of brown versus white adipose tissue in this study. Whether BAT or WAT localization or activation influences the severity of ALD should be explored.

ASH and ALD are complex physiological conditions that are influenced by metabolic activity, inflammation and the gut microbiome. Recreating, and probing the interplay of these systems should be central to enhancing our understandings of the mechanisms that drive alcohol-induced hepatic pathogenesis. The gut microbiome is heavily influenced by diet. Therefore, further studies should continue explore how diet and alcohol abuse in combination influence the composition of the gut microbiome and ALD pathology.

## Supporting information

S1 Fig Quantification of inflammation and fibrinogenesis in WASH-diet pair-fed control mice display. **A.** IHC expression of TGFB in vehicle and SR9238 treated WASH-control diet fed mice. **B.** IHC expression of IL6 in mice receiving the WASH-control diet and dosed with vehicle or SR9238 **C.** PicoSirius Red staining showing lack of hepatic collagen deposits in livers of WASH control mice treated with SR9238 or vehicle. Immunohistochemistry was performed as described in the materials and methods section.
(PDF)

## References

[1] Report—Alcohol-Attributable Fractions, US, Excessive Use. [cited 20 Nov 2020]. Available: https://nccd.cdc.gov/DPH_ARDI/Default/Report.aspx?T=AAF&P=1A04A664-0244-42C1-91DE-316F3AF6B447&R=B885BD06-13DF-45CD-8DD8-AA6B178C4ECE&F=&D=

[2] Publications | National Institute on Alcohol Abuse and Alcoholism | Surveillance Report #113. [cited 20 Nov 2020]. Available: https://pubs.niaaa.nih.gov/publications/surveillance113/CONS17.htm

[3] Kirpich IA, Miller ME, Cave MC, Joshi-Barve S, McClain CJ. Alcoholic liver disease: Update on the role of dietary fat. Biomolecules. MDPI AG; 2016. pp. 2–17. 10.3390/biom6010001 26751488 PMC4808795

[4] Chiang DJ, McCullough AJ. The impact of obesity and metabolic syndrome on alcoholic liver disease. Clinics in Liver Disease. NIH Public Access; 2014. pp. 157–163. 10.1016/j.cld.2013.09.006 PMC6130318 24274871

[5] Hwang S, Ren T, Gao B. Obesity and binge alcohol intake are deadly combination to induce steatohepatitis: A model of high-fat diet and binge ethanol intake. Clinical and Molecular Hepatology. Korean Association for the Study of the Liver; 2020. pp. 586–594. 10.3350/cmh.2020.0100 PMC7641546 32937687

[6] Chang B, Xu MJ, Zhou Z, Cai Y, Li M, Wang W, et al. Short- or long-term high-fat diet feeding plus acute ethanol binge synergistically induce acute liver injury in mice: An important role for CXCL1. Hepatology. 2015;62: 1070–1085. 10.1002/hep.27921 26033752 PMC4589443

[7] Dyson J, Jaques B, Chattopadyhay D, Lochan R, Graham J, Das D, et al. Hepatocellular cancer: The impact of obesity, type 2 diabetes and a multidisciplinary team. J Hepatol. 2014;60: 110–117. 10.1016/j.jhep.2013.08.011 23978719

[8] Jump DB, Depner CM, Tripathy S, Lytle KA. Impact of dietary fat on the development of non-alcoholic fatty liver disease in Ldlr-/- mice. Proceedings of the Nutrition Society. Cambridge University Press; 2016. pp. 1–9. 10.1017/S002966511500244X PMC4720541 26282529

[9] Hassan MM, Hwang LY, Hatten CJ, Swaim M, Li D, Abbruzzese JL, et al. Risk factors for hepatocellular carcinoma: Synergism of alcohol with viral hepatitis and diabetes mellitus. Hepatology. 2002;36: 1206–1213. 10.1053/jhep.2002.36780 12395331

[10] Everitt H, Hu M, Ajmo JM, Rogers CQ, Liang X, Zhang R, et al. Ethanol administration exacerbates the abnormalities in hepatic lipid oxidation in genetically obese mice. Am J Physiol—Gastrointest Liver Physiol. 2013;304: G38. 10.1152/ajpgi.00309.2012 23139221 PMC3543633

[11] Yamada K, Mizukoshi E, Seike T, Horii R, Kitahara M, Sunagozaka H, et al. Light alcohol consumption has the potential to suppress hepatocellular injury and liver fibrosis in non-alcoholic fatty liver disease. Aspichueta P, editor. PLoS One. 2018;13: e0191026. 10.1371/journal.pone.0191026 29342182 PMC5771612

[12] Bertola A, Mathews S, Ki SH, Wang H, Gao B. Mouse model of chronic and binge ethanol feeding (the NIAAA model). Nat Protoc. 2013;8: 627–637. 10.1038/nprot.2013.032 23449255 PMC3788579

[13] Guo F, Zheng K, Benedé-Ubieto R, Cubero FJ, Nevzorova YA. The Lieber-DeCarli Diet-A Flagship Model for Experimental Alcoholic Liver Disease. Alcohol Clin Exp Res. 2018;42: 1828–1840. 10.1111/acer.13840 30025151

[14] Brandon-Warner E, Schrum LW, Schmidt CM, McKillop IH. Rodent models of alcoholic liver disease: Of mice and men. Alcohol. Alcohol; 2012. pp. 715–725. 10.1016/j.alcohol.2012.08.004 22960051 PMC3496818

[15] Hao F, Cubero FJ, Ramadori P, Liao L, Haas U, Lambertz D, et al. Inhibition of Caspase-8 does not protect from alcohol-induced liver apoptosis but alleviates alcoholic hepatic steatosis in mice. Cell Death Dis. 2017;8: e3152. 10.1038/cddis.2017.532 29072704 PMC5680911

[16] Sengupta M, Griffett K, Flaveny CA, Burris TP. Inhibition of Hepatotoxicity by a LXR Inverse Agonist in a Model of Alcoholic Liver Disease. ACS Pharmacol Transl Sci. 2018;1: 50–60. 10.1021/acsptsci.8b00003 31696159 PMC6834356

[17] Griffett K, Welch RD, Flaveny CA, Kolar GR, Neuschwander-Tetri BA, Burris TP. The LXR inverse agonist SR9238 suppresses fibrosis in a model of non-alcoholic steatohepatitis. Mol Metab. 2015;4: 353–357. 10.1016/j.molmet.2015.01.009 25830098 PMC4354919

[18] Naveau S, Giraud V, Borotto E, Aubert A, Capron F, Chaput JC. Excess weight risk factor for alcoholic liver disease. Hepatology. 1997;25: 108–111. 10.1002/hep.510250120 8985274

[19] Stepanova M, Rafiq N, Younossi ZM. Components of metabolic syndrome are independent predictors of mortality in patients with chronic liver disease: A population-based study. Gut. 2010;59: 1410–1415. 10.1136/gut.2010.213553 20660697

[20] Loomba R, Bettencourt R, Barrett-Connor E. Synergistic association between alcohol intake and body mass index with serum alanine and aspartate aminotransferase levels in older adults: The Rancho Bernardo Study. Aliment Pharmacol Ther. 2009;30: 1137–1149. 10.1111/j.1365-2036.2009.04141.x 19737152 PMC3220929

[21] Shen H, Jiang L, Lin JD, Bishr Omary M, Rui L. Brown fat activation mitigates alcohol-induced liver steatosis and injury in mice. J Clin Invest. 2019;129: 2305–2317. 10.1172/JCI124376 30888335 PMC6546460

