# Supplementary material for: A two-hit model of alcoholic liver disease that exhibits rapid, severe fibrosis
Source: PLoS One. 2021 Mar 26;16(3):e0249316. doi: 10.1371/journal.pone.0249316 (PMC7996992; doi:10.1371/journal.pone.0249316)
Supplement: S1 Fig — A. IHC expression of TGFB in vehicle and SR9238 treated WASH-control diet fed mice. B. IHC expression of IL6 in mice receiving the WASH-control diet and dosed with vehicle or SR9238 C. PicoSirius Red staining showing lack of hepatic collagen deposits in livers of WASH control mice treated with SR9238 or vehicle. Immunohistochemistry was performed as described in the materials and methods section. (PDF) [file pone.0249316.s001.pdf]

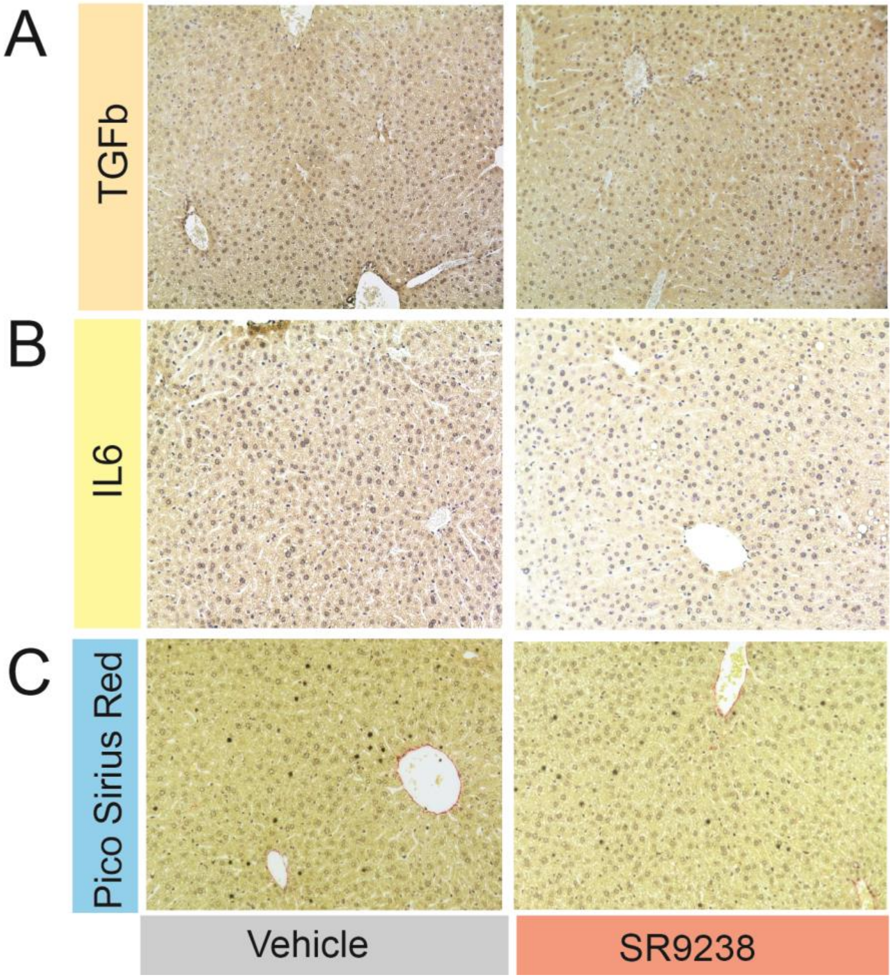

S1 Fig. Quantification of inflammation and fibrinogenesis in WASH-diet pair-fed control mice display. A. IHC expression of TGFB in vehicle and SR9238 treated WASH-control diet fed mice. **B.** IHC expression of IL6 in mice receiving the WASH-control diet and dosed with vehicle or SR9238 **C.** PicoSirius Red staining showing lack of hepatic collagen deposits in livers of WASH control mice treated with SR9238 or vehicle. Immunohistochemistry was performed as described in the materials and methods section.
